# Expression and characterization of a novel microbial GH9 glucanase, IDSGLUC9-4, isolated from sheep rumen

**DOI:** 10.5713/ab.24.0138

**Published:** 2024-05-29

**Authors:** Yongzhen Zhu, Shuning Bai, Nuo Li, Jun-Hong Wang, Jia-Kun Wang, Qian Wang, Kaiying Wang, Tietao Zhang

**Affiliations:** 1 Jilin Key Laboratory for Molecular Biology of Special Economic Animals, Institute of Special Animal and Plant Sciences, Chinese Academy of Agricultural Sciences, Changchun 130112, China; 2 Key Laboratory of Molecular Animal Nutrition, Ministry of Education, Zhejiang University, Hangzhou 310058, China; 3 Institute of Dairy Science, College of Animal Sciences, Zhejiang University, Hangzhou 310058, China

**Keywords:** Expression, β-Glucan, Glycoside Hydrolase, Hydrolysis, Rumen Microbiome

## Abstract

**Objective:**

This study aimed to identify and characterize a novel endo-β-glucanase, IDSGLUC9-4, from the rumen metatranscriptome of Hu sheep.

**Methods:**

A novel endo-β-glucanase, IDSGLUC9-4, was heterologously expressed in *Escherichia coli* and biochemically characterized. The optimal temperature and pH of recombinant IDSGLUC9-4 were determined. Subsequently, substrate specificity of the enzyme was assessed using mixed-linked glucans including barley β-glucan and Icelandic moss lichenan. Thin-layer chromatography (TLC), high-performance liquid chromatography (HPLC), matrix assisted laser desorption ionization time of flight mass spectrometry analyses were conducted to determine the products released from polysaccharides and cello-oligosaccharides substrates.

**Results:**

The recombinant IDSGLUC9-4 exhibited temperature and pH optima of 40°C and pH 6.0, respectively. It exclusively hydrolyzed mixed-linked glucans, with significant activity observed for barley β-glucan (109.59±3.61 μmol/mg min) and Icelandic moss lichenan (35.35±1.55 μmol/mg min). TLC and HPLC analyses revealed that IDSGLUC9-4 primarily released cellobiose, cellotriose, and cellotetraose from polysaccharide substrates. Furthermore, after 48 h of reaction, IDSGLUC9-4 removed most of the glucose, indicating transglycosylation activity alongside its endo-glucanase activity.

**Conclusion:**

The recombinant IDSGLUC9-4 was a relatively acid-resistant, mesophilic endo-glucanase (EC 3.2.1.4) that hydrolyzed glucan-like substrates, generating predominantly G3 and G4 oligosaccharides, and which appeared to have glycosylation activity. These findings provided insights into the substrate specificity and product profiles of rumen-derived GH9 glucanases and contributed to the expanding knowledge of cellulolytic enzymes and novel herbivore rumen enzymes in general.

## INTRODUCTION

Nonstarch polysaccharides (NSPs) in the plant cell wall, mainly cellulose and hemicellulose, make it challenging for animals to digest and absorb nutrients from plant-derived feedstocks. The presence of hemicellulose, one of the glucan-like NSPs in grain, acts as an anti-nutritional factor for monogastric animals, broiler chickens and pigs, resulting in increased chyme viscosity, and decreased feed utilization and growth performance [1,2]. Strategies for eliminating the anti-nutritional effects of glucan have been proposed, using biophysical and biochemical approaches [3], of which enzymic digestion has become the favored method to degrade glucan-like NSPs, thereby reducing their undesirable viscosity. Glucanases are a group of glycoside hydrolases (GH) responsible for breakdown of glucan-like substrates into mono- and oligo-saccharides. In the carbohydrate-active enzyme (CAZy) database (https://www.cazy.org/), glucanases are categorized into endo-acting enzymes (endo-β-1,4-glucanase, EC 3.2.1.4; endo-β-1,3[4]-glucanase [EC 3.2.1.6]; endo-β-1,3-glucanase/larminarinase [EC 3.2. 1.39]; endo-β-1,3-1,4-glucanase/lichenase [EC 3.2.1.73]; endo-β-1,6-glucanase [EC 3.2.1.75]) and exo-acting enzymes (cellobiohydrolase [EC 3.2.1.91 and EC 3.2.1.176], exo-β-1,4-glucanase/cellodextrinase [EC 3.2.1.74]), and β-glucosidase (EC 3.2.1.21) [4]. Endo-acting glucanases degrade their substrates by randomly cleaving internal glycosidic bonds within the main polysaccharide chain [5]. The anti-nutritional factor of undigestible glucan-like substrates is removed by glucanase digestion. More importantly, the polysaccharides are depolymerized into functional oligosaccharides, which are prebiotics for intestinal probiotic organisms, such as *Lactobacillus* sp. and *Bifidobacterium* sp. [6]. Therefore, the exploration and characterization of endo-β-1,4-glucanases (EC 3.2.1.4) has great importance for the development of feed and food additives.

Depending on differences in three-dimensional (3D) structure and catalytic mechanism, glucanases are allocated into the GH families 5, 6, 9, 10, 12, 16, 26, 44, 45, 64, 81, 128, 157, 158, and 162 in the CAZy database. The GH5 family includes many enzymes, which have essentially the same catalytic mechanism, and are sub-divided into 57 subfamilies [7,8]. In contrast to the well-studied GH5 family members, the catalytic activities and biochemical properties of GH9-derived glucanases, which have an inverting catalytic mechanism, remain to be elucidated. Over the past decade, a few GH9 glucanase, such as Umcel9y-1 from the rice paddy soil microbial metagenome [9], ChCHU_0961B from *Cytophaga hutchinsonii* [10] and PcMulGH9 from *Paenibacillus curdlanolyticus* [11], have been isolated and functionally characterized.

The herbivore rumen microbiome has garnered significant research interest due to its efficient digestion of cellulosic materials and its potential as a source of valuable CAZymes for industrial applications [12]. Exogenous enzyme preparations are vital in poultry and swine production, improving nutrient utilization efficiency, mitigating the effects of unwanted components, expanding feed ingredient options, and enhancing formulation accuracy. They also contribute to environmental sustainability by reducing excreta moisture content, addressing various issues, and promoting intestinal health and immunity. Glucanases in these preparations hydrolyze polysaccharides into oligosaccharides, acting as prebiotics for intestinal microorganisms. Extracts from rumen microorganisms offer a valuable resource for discovering novel glucanases. This study focuses on leveraging these resources by expressing novel glucanase genes, exploring their enzymatic properties, deepening our understanding of these enzymes, and laying the foundation for the development of feed enzyme preparations. In this study, a novel GH9 glucanase gene, *IDSGLUC9-4*, was identified from sheep rumen microbes using previously obtained transcriptomic data [13]. The gene was expressed heterologously in *Escherichia coli*, and the substrate specificity and product profile of the recombinant enzyme were characterized using a variety of glucan-like substrates.

## MATERIALS AND METHODS

### Materials

The gene, *IDSGLUC9-4* (GenBank accession no. WUV 41754.1), was annotated using metatranscriptomic data obtained previously [13], then the sequence was codon optimized and synthesized by GenScript (Nanjing, China). *Escherichia coli* (*E. coli*) BL21(DE3) and the expression vector pET30a(+) were produced in our laboratory [14]. The substrates, barley β-glucan (CAS Number 9041-22-9; purity>95%), Icelandic moss lichenan (CAS Number 1402-10-4; purity>75%), tamarind xyloglucan (CAS Number 37294-28-3; purity>95%), konjac gum glucomannan (CAS Number 11078-31-2; purity >98%), cellotriose (CAS Number 33404-34-1; purity>95%), cellotetraose (CAS Number 38819-01-1; purity>90%) and cellopentaose (CAS Number 2240-27-9; purity>95%) were from Megazyme (Wicklow, Ireland), cellobiose (CAS Number 528-50-7; purity>98%) was from Sigma-Aldrich (Darmstadt, Germany). Culture media, kanamycin, 6× His-tagged Ni-NTA resin and isopropyl-thio-β-D-galactopyranoside (IPTG) were from Sangon Biotech (Shanghai, China).

### Sequence analysis

Isoelectric point and molecular weight (pI, Mw) were predicted using the Expasy online tool (https://web.expasy.org/compute_pi/). Signal peptide and functional domain prediction were performed using InterPro (https://www.ebi.ac.uk/interpro/). SWISS-MODEL was employed for comparative modeling of tertiary structures (https://swissmodel.expasy.org/interactive/). Multiple sequence alignment and phylogenetic analysis were conducted using MEGA software (v.11.0; Kumer Lab, Temple University, Philadelphia, PA, USA). Phylogenetic analysis used the Maximum Likelihood statistical method, employing the WAG correction model. The phylogeny assessment used the bootstrap method with 500 bootstrap replications. MEGA and SWISS-MODEL were used for this analysis.

### Protein expression and purification

The transformation of the plasmid pET30a-*IDSGLUC9-4* was carried out by heat shocking it into *E. coli* BL21(DE3) competent cells and streaking the mixture onto Luria-Bertani (LB) plates (5 g/L yeast extract, 10 g/L tryptone, 10 g/L sodium chloride, 18 g/L agar, 100 μg/mL kanamycin). Colonies containing the *IDSGLUC9-4* gene were confirmed through colony-PCR and plasmid sequencing, and designated as BL21/pET30a-*IDSGLUC9-4*. The recombinant strain was added to LB medium (500 mL) with a 1% inoculum, then cultured at 37°C with continuously shaking at 180 rpm until the OD_600_ reached 0.6 to 1.0. Then, IPTG (250 μL, 1 mol/L) was added to induce protein expression at 16°C with gentle agitation at 100 rpm for 16 h. The cultured cells were harvested by centrifugation at 4°C and 6,000×*g* for 10 min, then resuspended in 1× phosphate-buffered saline (50 mL). A 15-min sonication at 65% power, with a 4 s pause every 2 s, was performed using an ultrasonic disruptor (TZL-1200; Perwell, Suzhou, China) to yield the crude enzyme. The crude enzyme solution was treated with Affinity Ni-NTA agarose resin at a ratio of 1:50, then supplemented with 1 mL of 1 mol/L imidazole. Following this, the mixture was gently agitated at 100 rpm for 1 hour at 0°C. The mixture was gently loaded into a chromatography column and the target protein was eluted stepwise with phosphate buffer, containing 20, 50, or 250 mmol/L imidazole. The purified protein was used for sodium dodecyl sulfate polyacrylamide gel electrophoresis (SDS-PAGE), and analysis of substrate selectivity and product profile. The enzyme substrate selectivity was tested on agar plates containing 0.2% (w/v) barley β-glucan, Icelandic moss lichenan, or konjac gum. Approximately 20 U of enzyme activity was spotted onto the plates, which were incubated for 16 h at 25°C, stained with a 0.1% (w/v) Congo red solution for 20 min, then destained with 1 mol/L NaCl until the clear areas became visible.

### Enzyme characterization

Enzyme activity was assessed by the 3,5-dinitrosalicylic acid (DNS) reducing sugar assay [15], and the protein concentration was determined using the Bradford assay [16]. One unit (U) of glucanase was defined as the amount of enzyme needed to generate 1 μmol of reducing sugar per min. Briefly, enzyme solution (40 μL, ~0.32 μg) and substrate (40 μL, 5 mg/mL of barley β-glucan, Icelandic moss lichenan, locust bean gum, or konjac glucomannan), were mixed and reacted for 15 min under the optimum conditions (40°C, pH 6.0), then the amount of reducing sugar produced was quantified with DNS; all assays were performed in triplicate.

#### Optimum temperature determination

Enzyme solution (40 μL, ~0.32 μg) was mixed with barley β-glucan solution (40 μL, 0.5% w/v) and the reaction was carried out at different temperatures (20°C, 30°C, 40°C, 50°C, 60°C, 70°C, and 80°C) for 15 min, then DNS solution (80 μL) was added and incubated at 95°C for 10 min. After cooling to room temperature, the OD540 was determined using a microplate reader. In the control assay, deactivated enzyme solution (40 μL) was used, all other conditions remaining constant. The temperature corresponding to the maximum observed activity was designated as 100% and the relative activity at different temperatures was calculated.

#### Optimum pH

Enzyme solution (40 μL, ~0.32 μg) was mixed with barley β-glucan solution (40 μL, 0.5% w/v) and the reaction was carried out in buffer solutions with pH ranging from 3.0 to 10.0 (pH 3.0, 4.0, 5.0, 6.0, 7.0 8.0, citrate/phosphate buffer; pH 8.0, 9.0,Tris-HCl buffer; pH 9.0 to 10.0, Glycine-NaOH buffer) and reacted for 15 min at 40°C. The pH of maximum activity was designated as 100%, and the relative activity at different pH values was calculated.

#### Thermal stability

The enzyme solution was incubated at 30°C, 40°C, 50°C, or 60°C for 5, 10, 30, or 60 min, then an aliquot (40 μL) was mixed with barley β-glucan in citric acid/phosphate buffer (40 μL, 0.5%, pH 6.0), and reacted for 15-min at 40°C. The activity of the non-heat-treated enzyme was designated as 100%, and the residual activity at each temperature was calculated.

#### The pH stability

Enzyme solution was mixed in a 1:1 ratio with buffer solutions ranging from pH 3.0 to 10.0 (pH 3.0, 4.0, 5.0, 6.0, 7.0 8.0, citrate/phosphate buffer; pH 8.0, 9.0, Tris-HCl buffer; pH 9.0 to 10.0, glycine-NaOH buffer), chilled on ice for 60 min, then an aliquot (40 μL) was mixed with barley β-glucan solution (40 μL, 0.5% w/v, pH 6.0) and reacted for 15 min at 40°C. The untreated enzyme activity was designated as 100%, and the residual activity at each pH value was calculated.

#### Effects of metal ions and organic reagents on glucosidase activity

Recombinant glucosidase solution was mixed with solutions of different concentrations of various metal chlorides (KCl, NaCl, CaCl_2_, MgCl_2_, ZnCl_2_, CuCl_2_, MnCl_2_, NiCl_2_), or mixed with different concentrations of organic solvents (ethylene diamine tetraacetic acid [EDTA], SDS, methanol, ethanol, propanol, butanol, dimethyl sulfoxide [DMSO]). After gentle centrifugation and mixing, the mixture was placed on ice for 30 minutes (metal chloride solution concentrations set at 1 mM or 5 mM, organic solvent concentrations set at 10 mM or 20 mM). The residual enzyme activity was determined under the influence of different metal ions and organic solvents, and calculated by comparison to the enzyme solution with only buffer added, which was considered 100%.

### Hydrolysis product profile determination

Enzyme solution (3 mL, ~24 μg) was combined with polysaccharide substrate solution (6 mL; 5 mg/mL of barley β-glucan, Iceland moss lichenan; 0.25 mg/mL of oligosaccharides, G1, glucose; G2, cellobiose; G3, cellotriose; G4, cellotetraose; G5, cellopentaose) and incubated at 37°C. Samples were collected at 0, 20 s, 5, 10, 20, and 30 min, and 1, 3, 6, 12, 24, and 48 h; the reaction was terminated by heating at 95°C for 15 min and the samples centrifuged at 4°C and 10,000×g. The supernatant was used for product profile analysis in three parallel experiments. The hydrolysis products generated by IDSGLUC9-4 were analyzed by thin-layer chromatography (TLC) with n-butanol/acetic acid/water (5:2:3, v/v), as described previously [17], with an oligosaccharide standard mixture (G1–G5) for comparison.

A more in-depth analysis of the hydrolysis products was performed by high-performance liquid chromatography (HPLC), with an LC-1200 instrument (Agilent Technologies, Santa Clara, CA, USA), fitted with an Asahipak NH2P-50 4E chromatography column (Shodex, Tokyo, Japan) and an RID-20A refractive index detector. The mobile phase was 65% aqueous acetonitrile, with a column temperature of 40°C and a flow rate of 1.0 mL/min. In addition, hydrolysis samples taken at 3 and 48 h were analyzed by Ultraflextreme MALDI-TOF/TOF (Bruker, Billerica, MA, USA).

### Statistical analysis

The experiments conducted in this study, including protein concentration determination, DNS colorimetric reaction in enzymatic property studies, and quantification of oligosaccharide concentration in HPLC experiments, were all performed in triplicate. Image preparation and data analysis, including standard deviation and mean calculation, were conducted using GraphPad Prism v.8.0 (San Diego, CA, USA). Data were presented as the mean±standard deviation (n = 3). The section on “Effects of Metal Ions and Organic Reagents on Glucosidase Activity” involved single-factor comparisons. Statistical significance was analyzed using the Student’s *t*-test integrated into GraphPad Prism (* p< 0.05; ** p<0.01; *** p<0.001). Significant differences were assessed by comparing each experimental group with the control group treated without inhibitors, with the control group set as 100%.

## RESULTS AND DISCUSSION

### Glucanase gene mining and heterologous expression

**Figure 1:**
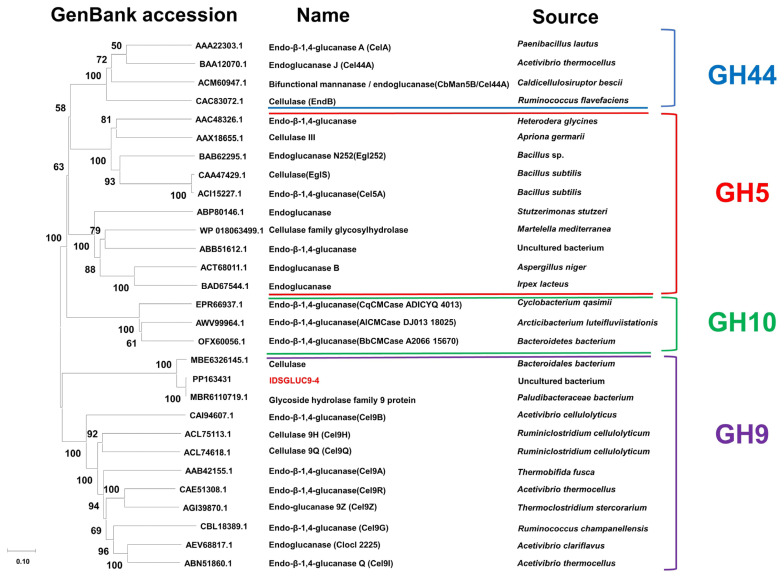
Phylogenetic analysis of IDSGLUC9-4 (GenBank accession no. WUV41754.1). Multiple sequence alignment was performed and used to generate a phylogenetic tree using MEGA11.0 and the maximum likelihood (ML) statistical method based on the WAG correction model, with a bootstrap of 500. The tree was drawn to scale (length = 0.1), and branch length was measured according to the number of substitutions per site.

**Figure 2:**
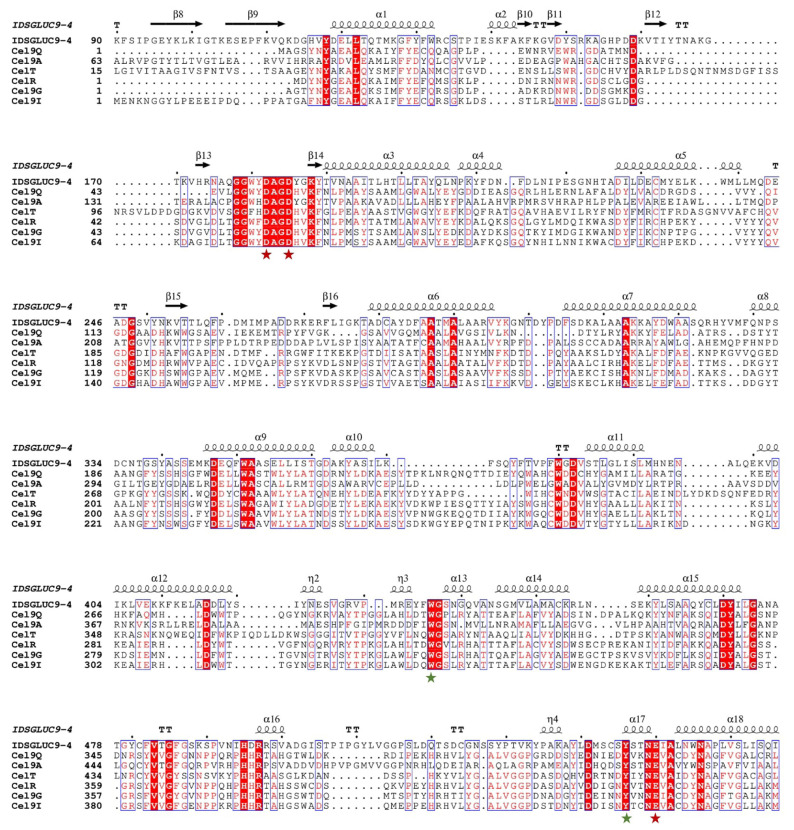
Multiple sequence alignment of IDSGLUC9-4 and six functionally-characterized GH 9 endoglucanases. The three catalytic residues and the conserved aromatic residues in the active site of GH9 glucanases are indicated by red and green asterisks, respectively. Cel9Q, from *Clostridium thermocellum* (PDB: 5GXX); Cel9A, from *Alicyclobacillus acidocaldarius* (PDB: 3EZ8); CelT, from *Clostridium thermocellum* (PDB: 2YIK); CelR, from *Acetovibrio thermocellus* (PDB: 7UNP); Cel9G, from *Clostridium thermocellum* (PDB: 1G87); Cel9I, from *Clostridium thermocellum* (PDB: 2XFG).

Ruminants, such as sheep and cattle, are unable to digest cellulose and other plant cell wall polysaccharides, so they obtain most of their nutrition indirectly from the action of cellulolytic microorganisms in their rumens [18]. Therefore, the rumen microbiome is a rich source of industrial CAZymes with high activity and useful properties. In this study, a GH9 β-glucanase gene, *IDSGLUC9-4* (GenBank accession no. WUV41754.1), was isolated from sheep rumen microbes, using transcriptomic data obtained previously [13]. The IDSGLUC9-4’s open reading frame spanned 1,716 bp, encoding 571 amino acids with a theoretical molecular mass of 63.6 kDa and an isoelectric point of 6.12. Multiple sequence alignment suggested that the *IDSGLUC9-4* gene shared high homology (>99%) with two other nucleotide sequences which annotated as GH family 9 protein and cellulase (GenBank accession no. MBR6110719.1 and MBE6326145.1), located in a metagenome-assembled genome annotated as *Paludibacteracea bacterium* and *Bacteroidales bacterium*. However, neither of these homologous enzymes has been functionally characterized. Phylogenetic analysis revealed that IDSGLUC9-4 and another enzyme (GenBank accession no. MBR6110719.1) from the *Paludibacteraceae* are both in the GH9 family (Figure 1). The catalytic mechanism of GH9 enzymes involves an inversion of anomeric stereochemistry. Cel9A, a processive endoglucanase from *Thermobifida fusca*, is active against bacterial cellulose and is the only known cellulase capable of independently degrading crystalline regions in bacterial cellulose, although it prefers amorphous regions [19]. A closely related cellulase from *Clostridium phytofermentans* is the only GH9 cellulase encoded in its genome and is essential for cellulose degradation by the organism. Notably, this is the only documented instance of a single cellulase being essential for growth on cellulose [20]. Detailed sequence alignment suggests that IDSGLUC9-4 and six other GH9 enzymes have five shared amino acid residues, three catalytic residues (Asp182, Asp184, and Glu560) and two conserved aromatic residues (Trp436 and Tyr545) (Figure 2) [21–24].

**Figure 3:**
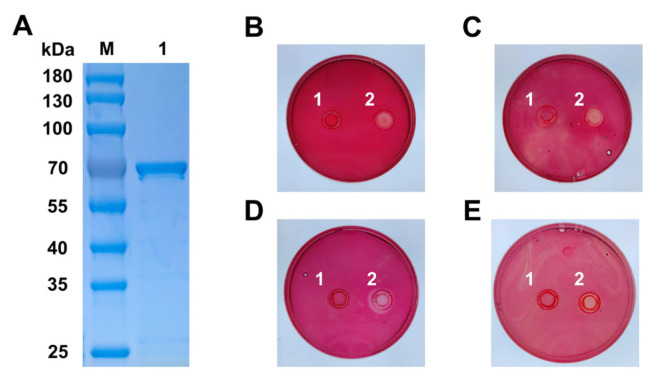
Purification and substrate selectivity determination of recombinant IDSGLUC9-4. (A) SDS-PAGE (M is standard protein marker, 1 is purified IDSGLUC9-4). Agar plates treated with BSA (1) and IDSGLUC9-4 (2), containing 0.2% polysaccharide substrate: barley β-glucan (B), Icelandic moss lichenan (C), xyloglucan (D), konjac gum (E). SDS-PAGE, sodium dodecyl sulfate polyacrylamide gel electrophoresis.

To investigate the biochemical properties of IDSGLUC9-4, the gene was expressed heterologously in *E. coli* to produce the recombinant enzyme. Following 6× His-tagged affinity purification, a prominent band at ~69 kDa was observed by SDS-PAGE (Figure 3A), consistent with its theoretical molecular weight of 63.4 kDa plus the expression vector backbone sequence of 5.3 kDa. Zymogram analysis indicated that IDSGLUC9-4 was active towards barley β-glucan, Icelandic moss lichenan, xyloglucan, and konjac gum (Figure 3B to 3E).

The GH9 family protein derived from the rumen of Hu sheep exhibits the capability to hydrolyze mixed-linkage glucans. Comparative analysis with previously characterized GH9 family glucanases reveals the presence of similar catalytic residues, indicating the successful isolation of a novel, uncharacterized glucanase from the sheep rumen. This enzyme represents a common exogenous enzyme preparation in livestock production. In-depth enzymatic and hydrolytic property studies will facilitate a comprehensive understanding of its functionality.

### Biochemical properties of recombinant IDSGLUC9-4

**Figure 4:**
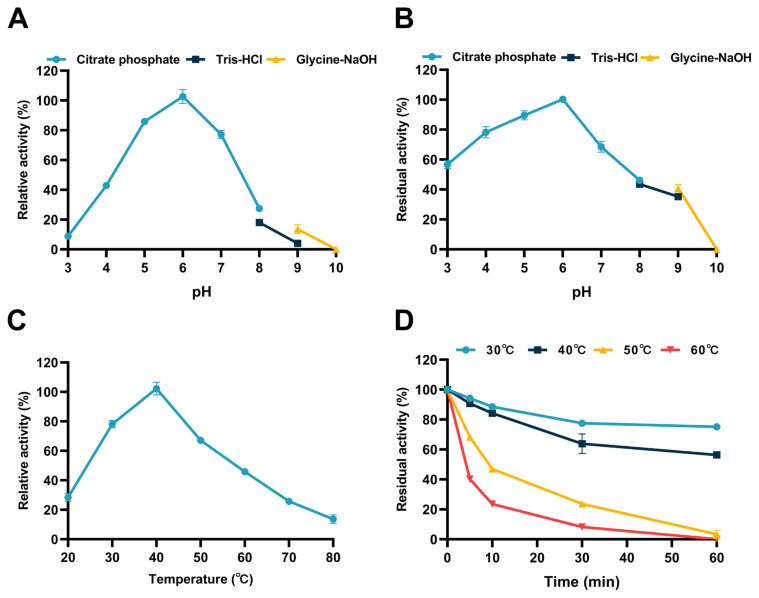
Enzymic properties of IDSGLUC9-4. (A) pH profile of enzyme activity. (B) pH stability. (C) Temperature profile of enzyme activity. (D) Thermostability. Data expressed as the mean±standard deviation (n = 3).

The optimum pH of IDSGLUC9-4 was 6.0, with >75% of the maximum catalytic activity between pH 5.0 to 7.0 (Figure 4A). IDSGLUC9-4 was relatively stable (> 70%) between pH 4.0 to 6.0 (Figure 4B) and most stable at pH 6.0, but relatively unstable above pH 7.0. Notably, after preincubation for 1 h at pH 4.0 and 5.0, the residual activities were 78.22% and 89.53%, respectively, indicating that the enzyme and its unknown producing microorganism were well-adapted to the acidic environment of the rumen [17,18]. The optimum temperature for IDSGLUC9-4 was 40°C (Figure 4C) and the enzyme was much less stable at higher temperatures. Thermostability assays revealed that the IDSGLUC9-4 was sensitive to heat-challenge; after 1 h preincubation at 30°C and 40°C, the enzyme retained 75.10%±0.43% and 56.33%±1.14% of its initial activity, respectively (Figure 4D), and above 50°C, the activity decreased rapidly. These findings suggested that IDSGLUC9-4 was a relatively acid-resistant and mesophilic enzyme, which is consistent with the properties of other gastrointestinal tract-derived CAZymes reported previously [25–28].

Substrate selectivity analysis revealed that IDSGLUC9-4 could hydrolyze barley β-glucan, Icelandic moss lichenan, konjac glucomannan and tamarind xyloglucan; barley β-glucan was the substrate with the highest catalytic activity, 109.59± 3.61 μmol/mg min (Table 1). IDSGLUC9-4 was inactive towards beechwood xylan, galactomannan, guar gum, arabinan, *Laminaria digitata* laminaran, locust bean gum and arabinoxylan. The effects of metal ions and organic compounds on the activity of the enzyme, with β-glucan as substrate, were also determined at 40°C (Table 2). All the metal ions inhibited the enzyme, with Mn^2+^ the strongest inhibitor, as did all the organic compounds, with propanol and methanol the strongest inhibitors. Previously reported β-glucanases have diverse behaviors under the influence of metal ions and organic compounds. The activity of an exo-β-1,3-glucanase from the moose rumen microbiome [29] more than doubled in the presence of zinc ions, retaining normal activity in the presence of EDTA, propanol, and butanol. In contrast, the activity of IDSGLUC9-4 slightly decreased in the presence of 20 mM EDTA (p<0.05) but lost >50% of its activity in the presence of DMSO and propanol.

Enzymatic property studies revealed that this novel glucanase exhibits a specific activity of 109.59±3.61 μmol/mg min towards β-glucan. It shows stability within the temperature range of 30°C to 40°C. Additionally, it demonstrates tolerance to acidity, maintaining over 75% activity after 1 hour of incubation at pH 4.0 to 5.0. These preferences for moderate temperature and tolerance to weak acidity align with its origin from the rumen environment. Moreover, its activity towards four different polysaccharide substrates suggests promising application potential for IDSGLUC9-4. Further investigation into its hydrolytic mechanism is required to comprehensively elucidate its enzymatic properties.

### Hydrolysis products released from polysaccharides and cello-oligosaccharides by IDSGLUC9-4

**Figure 5:**
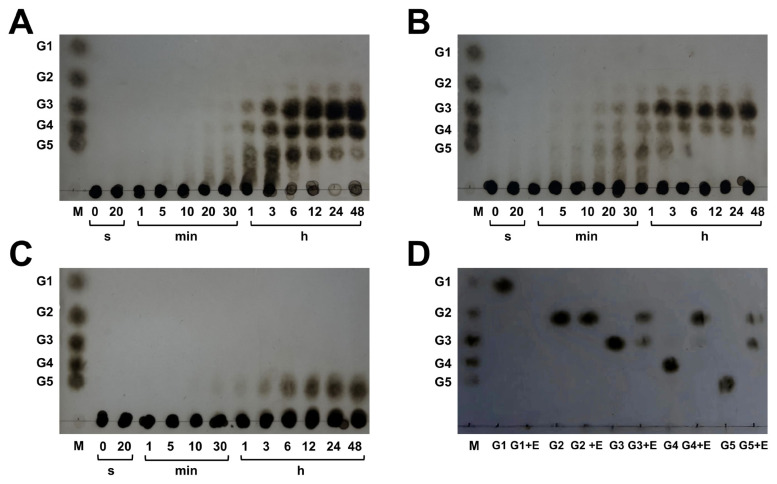
Hydrolytic products generated by IDSGLUC9-4 from glucan-like polysaccharides and cello-oligosaccharides analyzed by TLC. (A) barley β-glucan. (B) Icelandic moss lichenan. (C) Xyloglucan. (D) Cello-oligosaccharides. G1, glucose; G2, cellobiose; G3, cellotriose; G4, cellotetraose; G5, cellopentaose. TLC, thin-layer chromatography.

**Figure 6:**
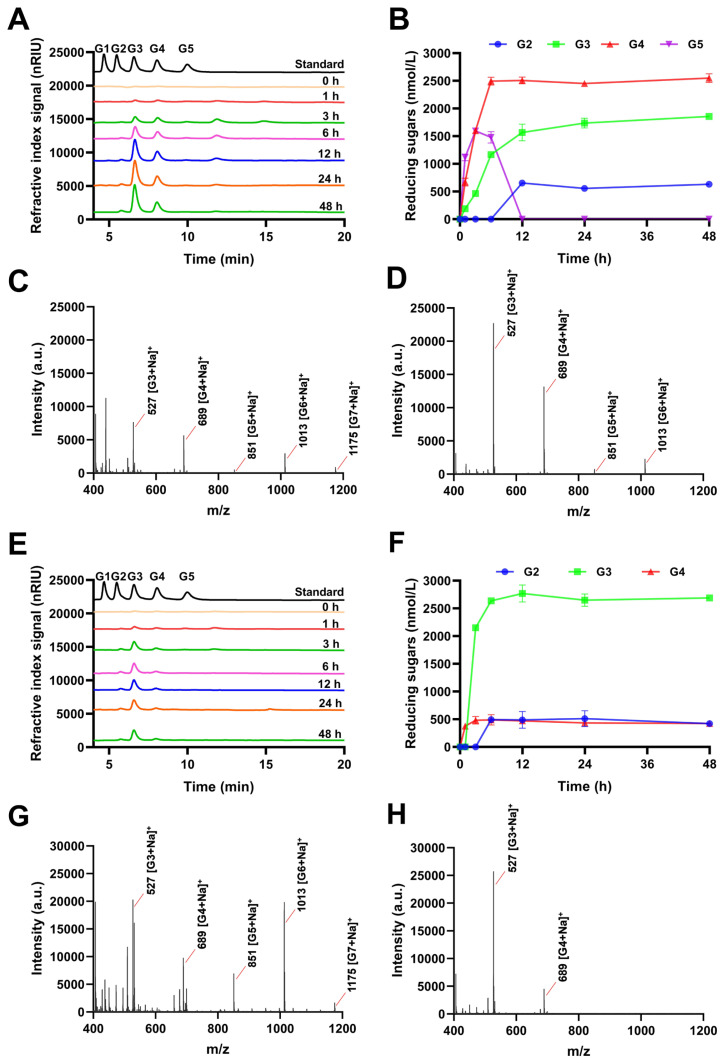
HPLC and MALDI-TOF analyses of the hydrolytic products of IDSGLUC9-4. (A) HPLC profiles (B); reducing sugar profile; (C) MALDI-TOF analysis at 3 h; (D) MALDI-TOF analysis at 48 h of hydrolyzed barley β-glucan. (E) HPLC profiles; (F) reducing sugar profile; (G) MALDI-TOF analysis at 1h; (H) MALDI-TOF analysis at 48 h of hydrolyzed Icelandic moss lichenan. Data expressed as mean±standard deviation (n = 3). G1, glucose; G2, cellobiose; G3, cellotriose; G4, cellotetraose; G5, cellopentaose. HPLC, high-performance liquid chromatography; MALDI-TOF, matrix assisted laser desorption ionization time of flight mass spectrometry.

**Figure 7:**
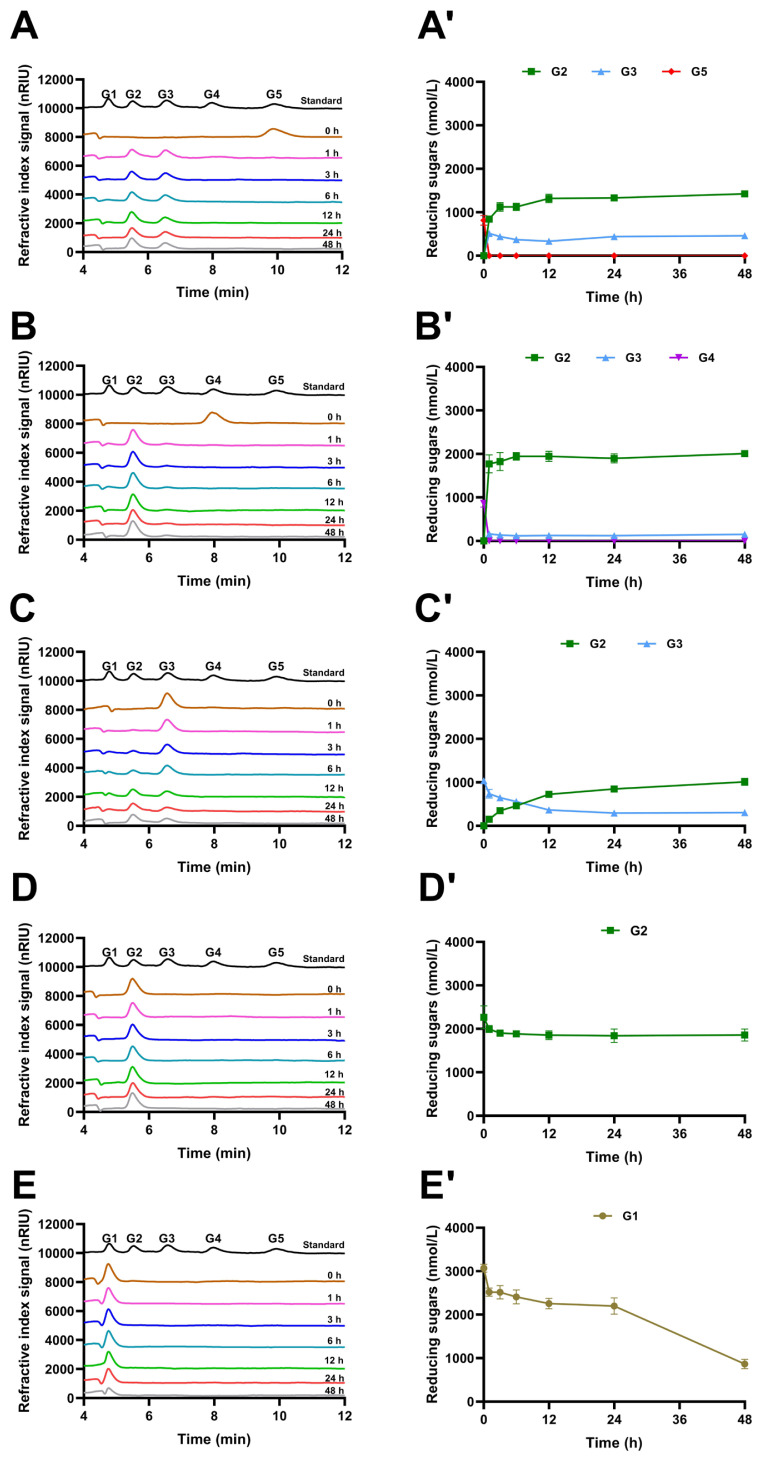
HPLC analyses of the hydrolytic product time-courses of IDSGLUC9-4. (A–E) HPLC profiles of G5 (A), G4 (B), G3 (C), G2 (D), and G1 (E). (A’–E’) Hydrolytic product profiles of G5 (A’), G4 (B’), G3 (C’), G2 (D’), and G1 (E’). Data are expressed as mean±standard deviation (n = 3). G1, glucose; G2, cellobiose; G3, cellotriose; G4, cellotetraose; G5, cellopentaose. HPLC, high-performance liquid chromatography.

To investigate the mechanism of action of IDSGLUC9-4, TLC, and HPLC were used to monitor the time-course profiles of oligosaccharides released from barley β-glucan, Icelandic moss lichen polysaccharide, tamarind xyloglucan, and oligosaccharides (Figures 5, 6, and 7). IDSGLUC9-4 initially liberated oligosaccharides with a degree of polymerization (DP) >5 from barley β-glucan and Icelandic moss lichenan; subsequently, these intermediates were further cleaved into smaller oligosaccharides as final products (Figures 5A, 5B, 6), indicating that IDSGLUC9-4 was an endo-acting glucanase (EC 3.2.1.4) [17]. IDSGLUC9-4 initially hydrolyzed barley β-glucan into G3, G4, and G5 (Figure 6); after 3 h of hydrolysis, the concentrations of G3, G4, and G5 constituted 1.10%, 49.53%, and 49.38% of total reducing sugars, respectively (Figures 6A, 6B). Matrix assisted laser desorption ionization time of flight mass spectrometry (MALDI-TOF) analysis at this time point confirmed the presence of G3–G7 and G9 (Figure 6C) and at longer times, the concentrations of G3 and G4 continued to increase, whereas G5 decreased with time., G2 was barely detectable up to 6 h, then its concentration rapidly increased up to 12 h, after which it was stable. IDSGLUC9-4 initially hydrolyzed Icelandic moss lichenan into G2, G3, and G4 (0 to 6 h); the product composition stabilized after 12 h and remained essentially constant until 48 h (Figures 6E, 6F). IDSGLUC9-4 produced mainly G2-G4 from Icelandic moss lichenan, whereas it produced G2–G5 from barley β-glucan. The total reducing sugar concentration after 48 h was 5,036.92±108.21 and 3,532.72±82.45 nmol/L from β-glucan and lichenan, respectively (Figures 6B, 6F). Mass spectrometry confirmed the initial presence of G3, G4, G5, G6, G7, and G9 during hydrolysis of both β-glucan and lichenan, whereas, after 48 h reaction, only the G3 and G4 oligosaccharides remained (Figures 7G, 6H). G3 and G4 were the main end-products from barley β-glucan, accounting for 36.86% and 50.56%, respectively, of the total reducing sugars, whereas G3 (53.37%) was the dominant end-product from Icelandic moss lichenan.

Although both barley β-glucan and Icelandic moss lichenan contain a mixture of β-1,3- and β-1,4-glycosidic linkages, the major products from IDSGLUC9-4 differed, probably because of different distributions of the β-1,3- and β-1,4-linkages within their main chains [27]. The β-glucan and lichen polysaccharide are mixed-linkage glucans (MLGs) with characteristic ratios of 1,4- to 1,3-β-D linkages. Considering the structure and product profile obtained from these MLG substrates, IDSGLUC9-4 appears to function as an endo-β-1,4-glucanase (EC 3.2.1.4), randomly cleaving β-1,4 linkages between glucose residues anywhere in the polysaccharide chain. HPLC detected the appearance of a peak around 12 min, corresponding to the presence of G6, with mass spectrometry detecting the production of higher DP oligosaccharides (G6, G7, G9) during the initial stage of the reaction. The concentrations of G2, G3, and G4 remained stable in both barley β-glucan and Icelandic moss lichenan reactions between 24 and 48 hours, indicating that the reaction had reached its endpoint. Notably, the enzyme had no activity against β-1,3-glucan, indicating its specificity for β-1,4-glycosidic linkages in its target substrates. This was very similar to the substrate specificity of the cellulases Cel5 and Cel5D from *Ruminococcus albus* [30]. IDSGLUC9-4 had a very low activity against tamarind xyloglucan, a mixed-linkage, highly branched glucan, consisting of xylose, galactose, arabinose and fucose [31]. IDSGLUC9-4, because of its specificity towards Glc1→4Glc linkages and inability to hydrolyse xylan or arabinan (Table 1), only cleaved tamarind xyloglucan into large oligosaccharides with DP >5 throughout the reaction (Figure 5C).

To further analyze the product profiles of IDSGLUC9-4, mono- and oligosaccharides were used as hydrolysis substrates (Figures 5D, 7D). TLC (Figure 5D) clearly showed that both G5 and G4 were effectively hydrolyzed by IDSGLUC9-4, generating G2 and G3 from G5 and G2 as the final product from G4. Quantitative determination by HPLC (Figures 7) showed that G5 was initially cleaved into equivalent amounts of G3 and G2, then the G3 concentration began to decrease at 1 h, whereas the G2 concentration increased, indicating hydrolysis of G3 into G2 (Figures 7A, 7A’). After 48 h, the products derived from G4 were G2 (2,007.05± 31.11 nmol/L) and G3 (151.40±5.13 nmol/L), whereas those from G5 were G2 (1,425.18±49.28 nmol/L) and G3 (461.40 ±40.38 nmol/L). IDSGLUC exclusively cleaved G4 into two molecules of G2 (Figures 7B, 7B’), but G2 was highly resistant to further hydrolysis; its concentration decreased only slightly from that at 3 h, even after 48 h (Figures 7D, 7D’). Hydrolysis of G3 into G2 (Figure 7C, 7C’) was incomplete and glucose was not detected, indicating that the glycosylation activity of the enzyme partially reversed the G3 hydrolysis and consumed all the glucose produced [9]. Notably, unlike the complete degradation of G4 and G5, the residual G3 concentration after 48 h was 304±15.27 nmol/L (29.3% of the initial amount), suggesting that IDSGlUC9-4 preferentially cleaves high-DP cello-oligosaccharides. In addition, glucose as substrate decreased from 3.070±0.085 to 0.866±0.014 (28.2% of the initial amount) after 48 h (Figure 7E and 7E’), indicating the formation of oligosaccharides and consistent with the glycosylation activity of IDSGlUC9-4.

Similarly, most characterized endo-glucanases hydrolyze polysaccharides into cello-oligosaccharides, such as G2, G3, G4, and G5 [17,32–34]. However, some glucanases have product profiles unlike normal endo-acting glucanases. For example, Cel5A-h38 from the sheep rumen has dual activities, i.e., endo-β-1,3-1,4-glucanase (EC 3.2.1.73) and exo-cellobiohydrolase (EC 3.2.1.91), and exclusively generates glucose and G2 from lichenan [27]. Another bifunctional glucanase/mannanase from *Prevotella* sp., IDSGH5-14, produces high-DP oligosaccharides from glucan- and mannan-like substrates [35]. In this study, IDSGlUC9-4 was active towards glucose (Figure 5D, 7E, and 7E’), apparently exhibiting glycosylation activity. Glycosylation activity is also commonly observed with β-glucosidases (EC3.2.1.21), which cleave alkyl- and aryl-β-glycosidic linkages, releasing glucose [9,36,37]; glycosylation may reduce feedback inhibition at high glucose concentrations. A recently reported GH3 aryl-β-glucosidase, GluLm, has not only normal β-glucosidase activity, but can also hydrolyze glycosylated phenolic compounds [37]. In the final stages of reactions using polysaccharides and higher molecular weight cello-oligosaccharides as substrates, various polymerization levels of cello-oligosaccharides coexist. This occurs even when compounds like G2, which can serve as final products in G3 and G4 reactions. Transglycosylation and hydrolysis may mutually influence each other, with transglycosylation potentially counteracting substrate hydrolysis as substrate concentrations increase. Initially, hydrolysis and transglycosylation occur at similar rates, producing glucose and oligosaccharides with higher DP. As the reaction progresses with increased substrate consumption, hydrolysis becomes predominant over transglycosylation. In the final phase, characterized by minimal substrate concentration, only hydrolysis is observed [38]. The modification of glycoside hydrolytic enzymes through glycine mutations, such as β-glucosidase, holds promise for advanced research in engineered microbes. This approach seeks to broaden substrate specificity and enhance transglycosylation activity. The glucotolerant β-glucosidase, BGL-1, derived from *Talaromyces amestolkiae*, shows lower catalytic efficiency in cello-oligosaccharide hydrolysis, potentially limiting its applicability in saccharification processes. The engineered glycosynthase variant, BGL-1-E521G, serves as a versatile tool for regioselective β-1,2 transglycosylation, displaying heightened efficiency in glycoside synthesis [39].

The analysis of the degradation mechanism of polysaccharides and oligosaccharides by IDSGLUC9-4 reveals its typical endo-glucanase activity, capable of hydrolyzing polysaccharide substrates containing β-1,4-glycosidic bonds to produce oligosaccharides with DP>2, with predominant hydrolysis products being G2, G3, and G4. Its action on fibrous oligosaccharides G1–G5 demonstrates the ability to hydrolyze oligosaccharides with DP≥3 into G2 and G3. The hydrolytic potential of polysaccharides and oligosaccharides suggests suitability as a feed enzyme preparation. It can hydrolyze cellulose, which is difficult for monogastric animals to digest, into oligosaccharides that are readily absorbable or utilizable by intestinal probiotics. The substrate spectrum of the enzyme includes β-glucan, lichenin, xylan, and laminarin, indicating its ability to degrade not only β-glucans with mixed linkages of 1,3–1,4 but also polysaccharides with other linkage forms. Future efforts may focus on enhancing the activity of recombinant glucanases through molecular evolution engineering, for application in the development of enzyme preparations for agricultural waste treatment or animal feed.

## CONCLUSION

The study identified a novel, uncharacterized GH9 family protein, IDSGLUC9-4, originating from the rumen of Hu sheep. Recombinant glucanase was heterologously expressed and functionally characterized, revealing it as an endo-glucanase with relative acid tolerance and preference for moderate temperatures. It exhibits activity towards various substrates including β-glucan, lichenan, xyloglucan, and konjac gum, completely hydrolyzing polysaccharides into oligosaccharides of G2, G3, and G4. This glucanase holds promising prospects as a feed enzyme preparation for monogastric animals, particularly in the hydrolysis and release of prebiotic oligosaccharides. Future investigations could explore enhancements through molecular evolution engineering, immobilization, and other methods to broaden its application potential in livestock production.

## Figures and Tables

**Table 1:** Polysaccharide substrate selectivity of IDSGLUC9-4^1)^

Substrate	Typical structures	Specific activity (μmol/mg min)
Barley β-glucan	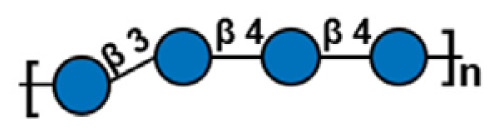	109.59±3.61
Icelandic moss lichenan	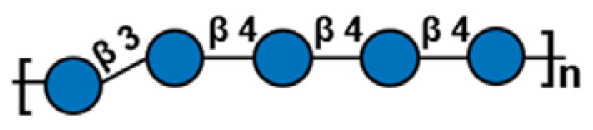	35.35±1.55
Konjac gum	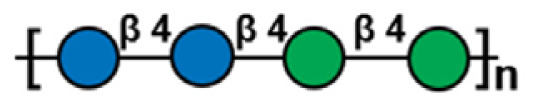	11.54±2.15
Tamarind xyloglucan	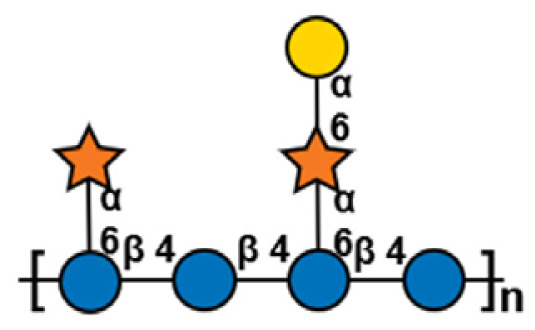	0.67±0.04
Beechwood xylan	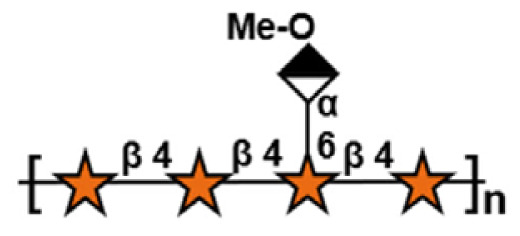	NA
Arabinan	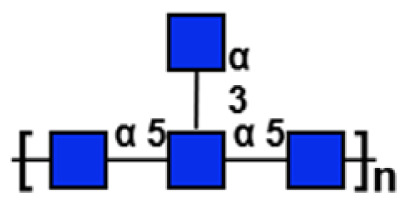	NA
Locust bean gum	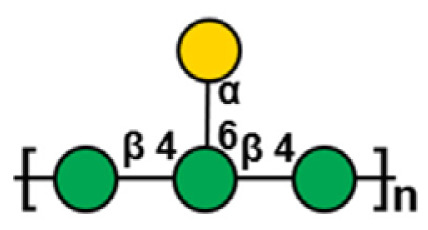	NA
Laminaria digitata laminaran	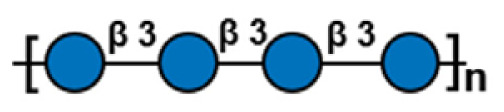	NA

1) Data expressed as mean±standard deviation (n = 3).

NA, not active.

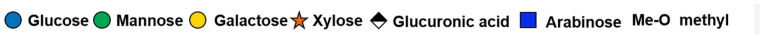

**Table 2:** Effect of metal ions and organic solvents on IDSGLUC9-4 activities^1)^

Metal ions	Residual activity (100%)		Organic reagents	Residual activity (100%)	
	
	1 mM	5 mM		10 mM	20 mM
NaCl	92.4±3.7^*^	86.3±2.9^**^	EDTA	95.6±4.5	89.0±2.3^**^
KCl	73.2±1.2^***^	61.6±0.5^***^	SDS	76.5±2.5^***^	64.3±2.4^***^
CaCl2	85.7±4.6^**^	56.1±4.8^***^	Propanol	46.6±4.7^***^	19.6±1.7^***^
MnCl2	19.2±0.6^***^	11.7±0.9^***^	Butanol	96.9±1.2^*^	75.5±4.1^***^
CuCl2	68.0±0.7^***^	49.7±2.1^***^	Methanol	56.2±2.6^***^	32.1±3.4^***^
NiCl2	57.3±0.5^***^	43.4±1.6^***^	Ethanol	74.3±3.7^***^	63.3±2.8^***^
MgCl2	87.4±1.0^***^	71.6±1.5^***^	DMSO	47.2±3.4^***^	38.9±1.5^***^
ZnCl2	95.5±2.2^*^	64.3±1.9^***^			

1) Data expressed as mean±standard deviation (n = 3).

EDTA, ethylene diamine tetraacetic acid; SDS, sodium dodecyl sulfate; DMSO, dimethyl sulfoxide.

The activity of IDSGLUC9-4 without treatment was designated as 100%. Single-factor comparisons were conducted between experimental and untreated control groups, with significant differences analyzed using the Student’s t-test (* p<0.05, ** p<0.01, *** p<0.00^1)^.
